# Supplementary material for: Early warning indicators of HIV drug resistance in the southern highlands region of Tanzania: Lessons from a cross-sectional surveillance study
Source: PLOS Glob Public Health. 2023 Mar 30;3(3):e0000929. doi: 10.1371/journal.pgph.0000929 (PMC10062592; doi:10.1371/journal.pgph.0000929)
Supplement: S1 Table — (DOCX) [file pgph.0000929.s001.docx]

**S1 Table: Details of facilities from which clients were enrolled**

| **No** | **Region** | **Name of health facility** | **Healthcare category *** | **Facility Ownership** | **Number of Pediatric Records Sampled** | **Number of Adult Records Sampled** | **Total Records Sampled** |
| --- | --- | --- | --- | --- | --- | --- | --- |
| 1 | Mbeya | St Bakhita Health Centre | Primary | Private | 25 | 405 | 430 |
| 2 | Mbeya | Madibira Health Centre | Primary | Government | 59 | 737 | 796 |
| 3 | Mbeya | Ruanda Health Centre | Primary | Government | 17 | 400 | 417 |
| 4 | Mbeya | Ilembo Health Centre | Primary | Government | 15 | 224 | 239 |
| 5 | Mbeya | Igawilo Health Centre | Primary | Government | 20 | 694 | 714 |
| 6 | Mbeya | Igogwe Mission Hospital | Primary | Private | 16 | 296 | 312 |
| 7 | Mbeya | Mbalizi Hospital (Ifisi) | Primary | Private | 17 | 321 | 338 |
| 8 | Mbeya | Matema Hospital | Primary | Private | 25 | 273 | 298 |
| 9 | Mbeya | Itete Hospital | Primary | Private | 17 | 260 | 277 |
| 10 | Mbeya | Mwakaleli Health Centre | Primary | Government | 14 | 238 | 252 |
| 11 | Mbeya | Mbeya Regional Referral Hospital | Secondary | Government | 34 | 626 | 660 |
| 12 | Mbeya | Chunya District hospital | Secondary | Government | 60 | 598 | 658 |
| 13 | Mbeya | Tukuyu District Hospital | Secondary | Government | 60 | 700 | 760 |
| 14 | Mbeya | Kyela District Hospital | Secondary | Government | 82 | 1076 | 1158 |
| 15 | Mbeya | Mbarali District Hospital | Secondary | Government | 59 | 737 | 796 |
| 16 | Mbeya | Mbeya Zonal Referral Hospital | Tertiary | Government | 120 | 625 | 745 |
| 17 | Mpanda | Town Clinic Health Centre | Primary | Government | 31 | 532 | 563 |
| 18 | Mpanda | Inyonga Health Centre | Primary | Government | 6 | 155 | 161 |
| 19 | Mpanda | Mamba Health Centre | Primary | Government | 8 | 144 | 152 |
| 20 | Mpanda | Mpanda District Hospital | Secondary | Government | 19 | 390 | 409 |
| 21 | Rukwa | Matai Health Centre | Primary | Government | 5 | 121 | 126 |
| 22 | Rukwa | Katandala Health Centre | Primary | Private | 5 | 154 | 159 |
| 23 | Rukwa | Mazwi Health Centre | Primary | Government | 15 | 390 | 405 |
| 24 | Rukwa | Dr.Artman Hospital | Primary | Private | 13 | 168 | 181 |
| 25 | Rukwa | Laela Health Centre | Primary | Government | 12 | 162 | 174 |
| 26 | Rukwa | Milepa Health Centre | Primary | Government | 20 | 299 | 319 |
| 27 | Rukwa | Mtowisa Health Centre | Primary | Government | 14 | 174 | 188 |
| 28 | Rukwa | Kirando Health Centre | Primary | Government | 12 | 181 | 193 |
| 29 | Rukwa | Namanyere District Hospital | Secondary | Government | 12 | 147 | 159 |
| 30 | Rukwa | Sumbawanga Regional Referral | Tertiary | Government | 35 | 274 | 309 |
| 31 | Ruvuma | Namtumbo Health Centre | Primary | Government | 4 | 141 | 145 |
| 32 | Ruvuma | Madaba Health Centre | Primary | Government | 9 | 110 | 119 |
| 33 | Ruvuma | Peramiho Hospital | Primary | Private | 25 | 164 | 189 |
| 34 | Ruvuma | St Annes Liuli Hospital | Primary | Private | 10 | 92 | 102 |
| 35 | Ruvuma | Mbamba Bay Health Centre | Primary | Government | 8 | 100 | 108 |
| 36 | Ruvuma | St Camillus Hospital | Primary | Private | 4 | 129 | 133 |
| 37 | Ruvuma | Mjimwema health Centre | Primary | Government | 25 | 266 | 291 |
| 38 | Ruvuma | Kigonsera Health Centre | Primary | Private | 11 | 119 | 130 |
| 39 | Ruvuma | Mapera Health Centre | Primary | Government | 5 | 116 | 121 |
| 40 | Ruvuma | Litembo Hospital | Primary | Private | 11 | 182 | 193 |
| 41 | Ruvuma | Tunduru District Hospital | Secondary | Government | 22 | 271 | 293 |
| 42 | Ruvuma | Mbinga District Hospital | Secondary | Government | 33 | 529 | 562 |
| 43 | Ruvuma | Songea Regional Referral Hospital | Tertiary | Government | 35 | 438 | 473 |
| 44 | Songwe | Isansa Health Centre | Primary | Government | 17 | 243 | 260 |
| 45 | Songwe | Kamsamba Health Centre | Primary | Government | 35 | 355 | 390 |
| 46 | Songwe | Tunduma Health Centre | Primary | Government | 41 | 710 | 751 |
| 47 | Songwe | Vwawa District Hospital | Secondary | Government | 97 | 1207 | 1304 |
| 48 | Songwe | Mwambani District Hospital | Secondary | Private | 47 | 447 | 494 |
| 49 | Songwe | Itumba District Hospital (Ileje DH) | Secondary | Government | 11 | 192 | 203 |
| 50 | Songwe | Isoko District Hospital | Secondary | Private | 4 | 55 | 59 |
|  |  |  |  |  | 1301 | 17367 | 18668 |

* Primary: Health centers; Secondary: District and Regional hospitals; Tertiary: Zonal Hospitals.
