# Supplementary material for: Early warning indicators of HIV drug resistance in the southern highlands region of Tanzania: Lessons from a cross-sectional surveillance study
Source: PLOS Glob Public Health. 2023 Mar 30;3(3):e0000929. doi: 10.1371/journal.pgph.0000929 (PMC10062592; doi:10.1371/journal.pgph.0000929)
Supplement: S2 Table — (DOCX) [file pgph.0000929.s002.docx]

**S2 Table:** individual facility performance for pediatric population

| **REGION** | **DISTRICT** | **Facility Name** | **EWI 1: On-time pill pick up** | **EWI 2: Retention on ART** | **EWI 3: ARV Pharmacy stock-out** | **EWI 4^1^: Dispensing Practices** | **EWI 4^2^: Prescribing Practices** |
| --- | --- | --- | --- | --- | --- | --- | --- |
| **Mbeya** | **Mbeya DC** | Ilembo hc | 2/4 (50%) | 13/15 (87%) | 12/12 (100%) | N/F | 0/4 (0%) |
|  |  | Mbalizi hosp If | 2/2 (100%) | 14/17 (82%) | 12/12 (100%) | N/F | 0/2 (0%) |
|  | **Mbeya CC** | Mzrh | 47/62 (76%) | 225/309 (73%) | 12/12 (100%) | 0/47 (0%) | 0/62 (0%) |
|  |  | Mrrh | 2/4 (50%) | 18/34 (53%) | N/F | N/F | 0/4 (0%) |
|  |  | Ruanda hc | 5/6 (83%) | 11/17 (65%) | N/F | N/F | 0/6 (0%) |
|  |  | Igawilo hosp | 1/4 (25%) | 16/20 (80%) | N/F | N/F | 0/4 (0%) |
|  | **Chunya DC** | Chunya dh | 5/10 (50%) | 40/60 (67%) | N/F | 0/3 (0%) | 0/10 (0%) |
|  | **Rungwe DC** | Tukuyu dh | 13/15 (87%) | 54/60 (90%) | 1/1 (8%) | 0/13 (0%) | 0/15 (0%) |
|  |  | Igogwe mission | 2/5 (40%) | 8/16 (50%) | 8/8 (66%) | 0/3 (0.0%) | 0/5 (0%) |
|  | **Kyela DC** | Kyela dh | 7/17 (41%) | 54/80 (68%) | 9/9 (75%) | 0/5 (0%) | 0/17 (0%) |
|  |  | Matema hosp | 7/12 (58%) | 9/21 (43%) | N/F | 0/9 (0%) | 0/12 (0%) |
|  | **Mbarali DC** | Mbarali dh | 5/9 (56%) | 42/54 (78%) | 11/11 (91.0%) | 0/7 (0%) | 0/9 (0%) |
|  |  | St.Bakhita hc | 7/11 (64%) | 19/24 (79%) | 4/4 (33%) | 0/7 (0%) | 0/11 (0%) |
|  |  | Madibira hc | 4/15 (27%) | 13/19 (68%) | 5/5 (41%) | 0/10 (0%) | 0/15 (0%) |
|  | **Busokelo DC** | Itete hosp | 4/8 (50%) | 14/17 (82%) | 11/11 (91%) | 0/2 (0%) | 0/8 (0%) |
|  |  | Mwakaleli hc | 11/13 (85%) | 11/14 (79%) | 12/12 (100%) | 0/10 (0%) | 0/13 (0%) |
| **Songwe** | **Mbozi DC** | Vwawa dh | 11/13 (85%) | 69/90 (77%) | 12/12 (100%) | 0/10 (0%) | 0/13 (0%) |
|  |  | Isansa hc | 3/8 (38%) | 12/19 (63%) | 1/1 (8%) | 0/6 (0%) | 0/8 (0%) |
|  | **Momba DC** | Kamsamba hc | 8/10 (80%) | 26/35 (74%) | 10/10 (83%) | 0/9 (0%) | 0/10 (0%) |
|  | **Tunduma TC** | Tunduma hc | 6/11 (55%) | 30/41 (73%) | N/F | 0/9 (0%) | 0/11 (0%) |
|  | **Songwe DC** | Mwambani ddh | 7/14 (50%) | 41/44 (93%) | 0/0 (0%) | N/F | 0/14 (0%) |
|  | **Ileje DC** | Itumba/Ileje dh | 1/6 (17%) | 6/8 (75%) | 0/0 (0%) | 0/2 (0%) | 0/6 (0%) |
|  |  | Isoko ddh | 2/4 (50%) | 4/4 (100%) | 12/12 (100%) | 0/2 (0%) | 1/4 (25%) |
| **Ruvuma** | **Songea MC** | Songea rrh | 9/10 (90%) | 30/35 (86%) | 4/4 (33%) | 0/10 (0%) | 0/10 (0%) |
|  | **Tunduru DC** | Tunduru dh | 8/14 (57%) | 20/22 (91%) | N/F | 0/12 (0%) | 0/14 (0%) |
|  | **Namtumbo DC** | Namtumbo hc | 1/3 (33%) | 3/4 (75%) | 1/1 (8%) | 0/1 (0%) | 0/3 (0%) |
|  | **Madaba DC** | Madaba hc | 9/9 (100%) | 7/9 (78%) | N/F | 0/6 (0%) | 0/9 (0%) |
|  | **Songea DC** | Peramiho hosp | 7/14 (50%) | 24/25 (96%) | N/F | 0/14 (0%) | 0/14 (0%) |
|  | **Nyasa DC** | St.anne's liuli | 5/6 (83%) | 7/8 (88%) | 6/6 (50%) | 0/7 (0%) | 0/6 (0%) |
|  | **Nyasa DC** | Mbambabay hc | 6/7 (86%) | 6/8 (75%) | 3/3 (25%) | 0/6 (0%) | 0/7 (0%) |
|  | **Songea MC** | St.camilius | 2/4 (50%) | 4/4 (100%) | 0/0 (0%) | 0/2 (0%) | 0/4 (0%) |
|  | **Songea MC** | Mjimwema hc | 7/13 (54%) | 14/18 (78%) | 11/11 (91%) | 0/12 (0.0%) | 0/13 (0%) |
|  | **Mbinga DC** | Kigonsera hc | 10/11 (91%) | 8/10 (80%) | N/F | 0/0 (0%) | 0/11 (0%) |
|  |  | Mapera hc | 3/3 (100%) | 4/5 (80%) | 5/5 (41%) | 0/2 (0%) | 0/3 (0%) |
|  | **Mbinga TC** | Litembo hosp | 6/8 (75%) | 8/12 (67%) | 5/5 (41%) | 0/5 (0%) | 0/8 (0%) |
|  |  | Mbinga dh | 12/17 (71%) | 29/36 (81%) | 12/12 (100%) | 0/2 (0%) | 0/17 (0%) |
| **Rukwa** | **Sumbawanga MC** | Sumbawanga rrh | 7/11 (64%) | 15/19 (79%) | 4/4 (33%) | 0/10 (0%) | 0/11 (0%) |
|  |  | Katandala hc | 2/2 (100%) | 6/9 (67%) | 7/7 (58%) | 0/1 (0%) | 0/2 (0%) |
|  |  | Mazwi hc | 7/7 (100%) | 13/17 (76%) | N/F | 0/7 (0%) | 0/7 (0%) |
|  |  | Dr.atiman hosp | 6/6 (100%) | 9/12 (75%) | N/F | 0/8 (0%) | 0/6 (0%) |
|  | **Sumbawanga DC** | Laela hc | 8/9 (89%) | 8/9 (89%) | 12/12 (100%) | 0/7 (0%) | 0/9 (0%) |
|  |  | Milepa hc | 1/10 (10%) | 16/20 (80%) | 4/4 (33%) | 0/0 (0%) | 0/10 (0%) |
|  |  | Mtowisa hc | 2/10 (20%) | 13/15 (87%) | 2/2 (16%) | 0/1 (0%) | 0/10 (0%) |
|  | **Nkasi DC** | Kirando hc | 3/3 (100%) | 7/11 (64%) | 9/9 (75%) | 0/2 (0%) | 0/3 (0%) |
|  |  | Namanyere ddh | 6/9 (67%) | 8/11 (73%) | 4/4 (33.0%) | 0/8 (0%) | 0/9 (0%) |
|  | **kalambo DC** | Matai hc | 2/2 (100%) | 4/5 (80%) | 0/0 (0%) | 0/2 (0%) | 0/2 (0%) |
| **Katavi** | **Mpanda TC** | Mpanda dh | 3/5 (60%) | 13/18 (72%) | 10/10 (83%) | 0/3 (0%) | 0/5 (0%) |
|  |  | Town clinic hc | 4/4 (100%) | 27/37 (73%) | 2/2 (16%) | 0/4 (0%) | 0/4 (0%) |
|  | **Mlele DC** | Inyonga hc | 2/4 (50%) | 6/6 (100%) | 3/3 (25%) | 0/2 (0%) | 0/4 (0%) |
|  | **Mpimbwe DC** | Mamba hc | 1/5 (20%) | 4/9 (44%) | 10/10 (83%) | 0/2 (0%) | 0/5 (0%) |

*-The colors reflect the WHO color codes for performance of each indicator in respective facilities (red-poor, amber- fair and green-desirable scoring)*

*-NF- Not found (data was missing at the health facility records).*

*-The denominators for all EWIs except EWI-3, are from the total number of CTC clients followed up for their first year of ARV treatment at respective facilities, for the year 2013 according to the sample size of each facility. The denominator for EWI-3 is 12, referring to the 12 months of the year 2013 in which ARV supply was assessed.*
