# Supplementary material for: Early warning indicators of HIV drug resistance in the southern highlands region of Tanzania: Lessons from a cross-sectional surveillance study
Source: PLOS Glob Public Health. 2023 Mar 30;3(3):e0000929. doi: 10.1371/journal.pgph.0000929 (PMC10062592; doi:10.1371/journal.pgph.0000929)
Supplement: Attachment — Submitted filename: Response to Reviewers 11th January 2023.docx [file pgph.0000929.s004.docx]

Date: 11^th^ January 2023

**To:** The Editor PLOS Global Public Health

Dear Sir/Madam,

**REF: PGPH-D-22-01203: EARLY WARNING INDICATORS AT FACILITIES IN THE SOUTHERN HIGHLANDS REGION OF TANZANIA: LESSONS FOR HIV DRUG RESISTANCE SURVEILLANCE**

I hereby respond to the issues that were raised by the reviewers to the manuscript as below in italics:

1. **There are 15 authors listed on this article but the author contributions are note listed at the close of the manuscript with the other disclosures. When returning the manuscript, please provide the information specific to the contributions of each author per the PLOS requirements:** The authors contributions have been provided as required. This is as below:
2. **Please send a completed 'Competing Interests' statement***:*

*"The authors have declared that no competing interests exist" has been added.*

3. **Please amend your detailed Financial Disclosure statement. This is published with the article. It must therefore be completed in full sentences and contain the exact wording you wish to be published.**
   *The study was funded by The US President’s Emergency Plan for AIDS Relief (PEPFAR) under the cooperative agreement* Cooperative Agreement # W81XWH-11-2-0174*. “The funders had no role in study design, data collection and analysis, decision to publish, or preparation of the manuscript.”*
4. **We notice that your supplementary tables are included in the manuscript file. Please remove them and upload them with the file type 'Supporting Information'. Please ensure that each Supporting Information file has a legend listed in the manuscript after the references list:** *This has been addressed. A file with the name has been created and the supplemental tables moved there.*
5. **Please provide the completed STROBE checklist for cross-sectional studies (https://www.equator-network.org/reporting-guidelines/strobe/) with the page and line numbers for each reporting element noted in the checklist. Please make sure to review the supporting document with the criteria and rationale for each element. There are multiple elements not reported or partially reported in the current manuscript.**

*Thank you, we have completed the STROBE Checklist as requested and made any necessary updates in tracked changes throughout the manuscript where referenced in the checklist according to page and line number.*

6. **In addition, the dates for the data collection of the data used for the current study are not clearly presented. In the abstract, the data underlying this study was stated as "In 2016, we retrospectively collected EWI data from 50 CTCs for the January to December 2013 period." Then, the manuscript states, "From July 2016 to August 2018, trained data clerks and CTC nurses abstracted data from January – December 2013 for EWIs 1-4 (EWI-1 On-time pill pick-up; EWI-2 Retention on ART; EWI-3 Pharmacy ARV stock out; EWI-4 Pharmacy Dispensing practices; and EWI-4 Pharmacy prescribing practices)." The data for this study was not collected from 2016 to 2018, instead the data was abstracted. As such, the data is nearly ten year old. For this reason, the relevancy of the data needs to be questioned in 2022. Please explain how this data is relevant. When responding to the editor and reviewer feedback, please provide detailed information about other studies, if any, that were published with this data.** The data that was abstracted was the one entered into health records at study facilities from January to December 2013. This being an early warning indicator study that serves to highlight the impact of improper practices that can result in development of HIV drug resistance in the general population many years later. As such, this study and its findings are relevant and can be used to improve healthcare outcomes in health facilities.
7. **P. 6 Line 42. In this manuscript you provide an important rationale for routine EWI as it relates HIVDR and your research confirms this. However, in the abstract the language does not convey the urgency of implementing WHO EWIs. Suggested change: There is an urgent need to implement WHO EWI to minimize the emergence of preventable HIV drug resistance and to maintain the effectiveness of first- and second-line ART regimens. This is particularly critical in the context of new ART drug roll-out such as dolutegravir, during the COVID-19 pandemic when resultant HIV service disruptions require careful monitoring, and for virologic suppression as countries move closer to epidemic control.** This has been corrected and information added on line 41-46.
8. **In discussion, please indicate how this study aligns with WHO Global Strategy to further strengthen the significance of this study. This is taken from the strategy statement: “WHO has developed a Global Strategy for HIV Drug Resistance Prevention and Assessment using a public health approach (3). This strategy supports optimal functioning of treatment programmes to minimize emergence of preventable HIV drug resistance and to maintain the effectiveness of first- and second-line ART regimens. WHO recommends that countries adapt and implement a national strategy, based on the Global Strategy, to assess and minimize emergence of preventable HIV drug resistance.”** A statement has been added to the discussion as advised. This is on line 314-322.
9. **On P7.Line 82, you state: ‘To help protect these hard-won gains, the first national retrospective EWI study took place in 2010’. Can you please include more information about whether this has continued. If not then this is an opportunity to directly call on the TZ government and others to recommit efforts to routine EWI**. No other EWI has been done in Tanzania hence the study was timely and there is need for this to be done routinely. This information has been added to the paper. This has been added on line 87-89.
10. **Can you tell us in the text why you selected January – December 2013 as opposed to a more recent period? Is the age of the data a limitation that needs to be addressed in the limitations section?** Our original study start date was planned for 2015. We got approval for the study protocol in 2016. Each patient identified in 2013 had to be followed up for 12 complete months. This would make a patient that had started treatment on 31st December 2013 to have their complete 1 year follow-up up until 30th Dec 2014. Thus, the 2014 cohort would not be feasible if the study was to start in 2015. We agree that the age of the data is a limitation for generalizability and have further detailed this in the limitations. However, we also feel that this study still affords relevance because it helps to provide indication of possible development of HIV drug resistance in the population due to health facility practices carried out in the past.
11. **In the intro or discussion, can you provide more details about frequency of conducting EWI. What does WHO recommend? Do you agree? In the absence of a national strategy what would you suggest for others outside of TZ?** WHO recommends yearly monitoring of EWIs of HIVDR at all ART clinics or a representative sample of all health facilities in a specific country. We agree that monitoring of EWIs should be done but not on a yearly basis as it takes time to carry it out and it is also important to put corrective measures in place, monitor their implementation before doing another EWI. Nationally, we would advise it to be done every 3-5 years. In the absence of a national strategy, then countries can adopt facility-based monitoring. Health facilities can include this activity in their quality assurance procedures and conduct EWI as part of their performance improvement plans. This information has been added in the discussion section.

Yours faithfully.

Dr Samoel Ashimosi Khamadi

Corresponding author.
